# Cecal bascule – An unusual cause of intestinal obstruction: A case report

**DOI:** 10.1016/j.ijscr.2021.105888

**Published:** 2021-04-14

**Authors:** S. Areesha Shakeel, Junaid Zaman, Hamza Haroon

**Affiliations:** a Department of General Surgery, Patel Hospital, Karachi, Pakistan; b Department of General Surgery, Jinnah Postgraduate Medical Centre, Karachi, Pakistan

**Keywords:** Cecal bascule, Cecal volvulus, Case report, Cecectomy, Laparotomy

## Abstract

**Introduction:**

Cecal bascule is a rare type of cecal volvulus occurring in the elderly population with a male predominance. It is a condition where the cecum folds upward upon itself.

**Importance:**

There are only a handful of case reports published about cecal bascule. This is the first case being reported from Pakistan.

**Case presentation:**

A 68 year old female, known hypertensive and osteoporosis presented in the ER with complaints of vomiting, abdominal distension and absolute constipation. Radiology was non-diagnostic. She was treated with cecectomy and anastomosis. Post operatively she developed ileus for which was managed. She was subsequently discharged at 8th day post op.

**Clinical discussion:**

The management of cecal bascule is always surgery with predilection towards cecopexy or cecetomy to prevent recurrence.

This manuscript is as per the SCARE case report checklist [1].

## Highlights

• Cecal bascule is rare.
• Case of Elderly female with cecal bascule.
• Ceceomy + ileo-cecal anastomosis was done.
• Should consider cecal bascule in acute abdomen.

## 1 Introduction

Cecal volvulus is a well-defined term since the 1830s [2]. It is caused by the axial rotation of cecum, distal ileum and proximal colon, usually presenting in individuals who do not have normal cecal fixation [3]. A rare type of cecal volvulus is the cecal bascule in which the cecum may fold upward and anteriorly upon itself without an axial twist [[3], [4], [5]]. Cecal bascule has an incidence of 2.8–7.1 per million people per year, and makes up for only 1–2% of all large bowel obstructions [6]. There are only a handful of case reports about cecal bascule. Here, we present a case of cecal bascule, encountered in an elderly female which is the first case of cecal bascule being reported in Pakistan.

## 2 Case presentation

**Fig. 1:**
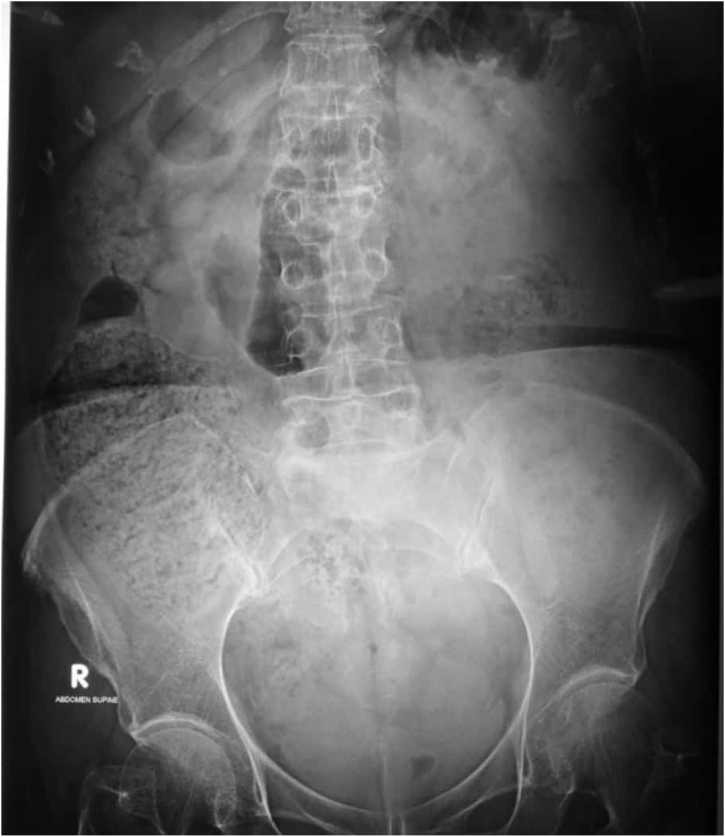
X-ray  abdomen supine.

**Fig. 2:**
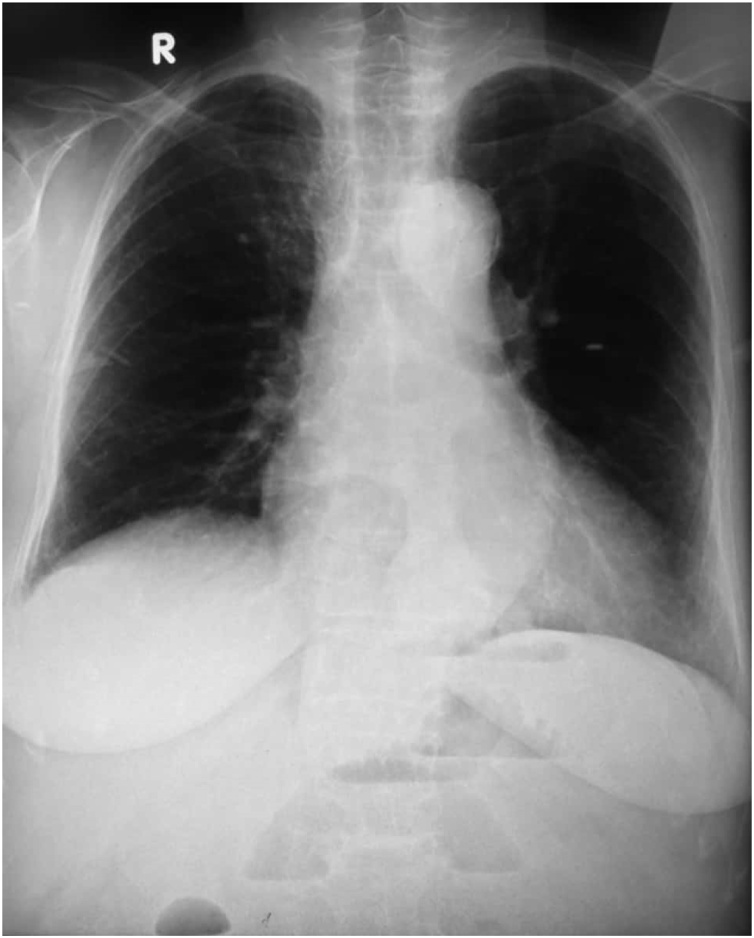
X-ray chest erect.

A 68 year old female, known case of hypertension and osteoporosis presented to the emergency department with complaints of absolute constipation for five days, abdominal distension for three days and vomiting for one day. Abdominal distention was gradual in onset and generalized. Vomitus was feculent and large in quantity. Apart from an uneventful caesarian section 35 years prior, she had no other past surgical history. On examination, the abdomen was tense, tympanatic and mildly tender on the right side on deep palpation. DRE revealed collapsed ano-rectal canal with no fecal staining. Her Xray abdomen supine shown below (Fig. 1) demonstrated dilated small bowel and cecal distension with fecal loading and no gas under the diaphragm was seen in the chest x-ray (Fig. 2). She had history for similar complaints one year back, for which she was managed conservatively. She was on medications for osteoporosis, and had family history of Chron’s disease.

**Fig. 3:**
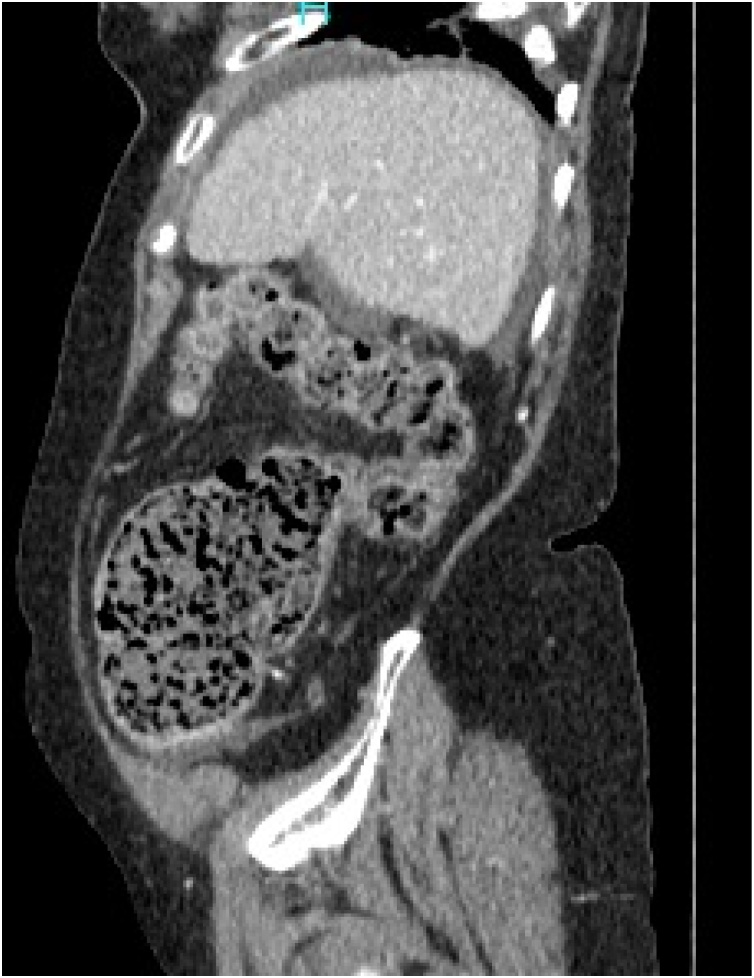
Sagittal view.

**Fig. 4:**
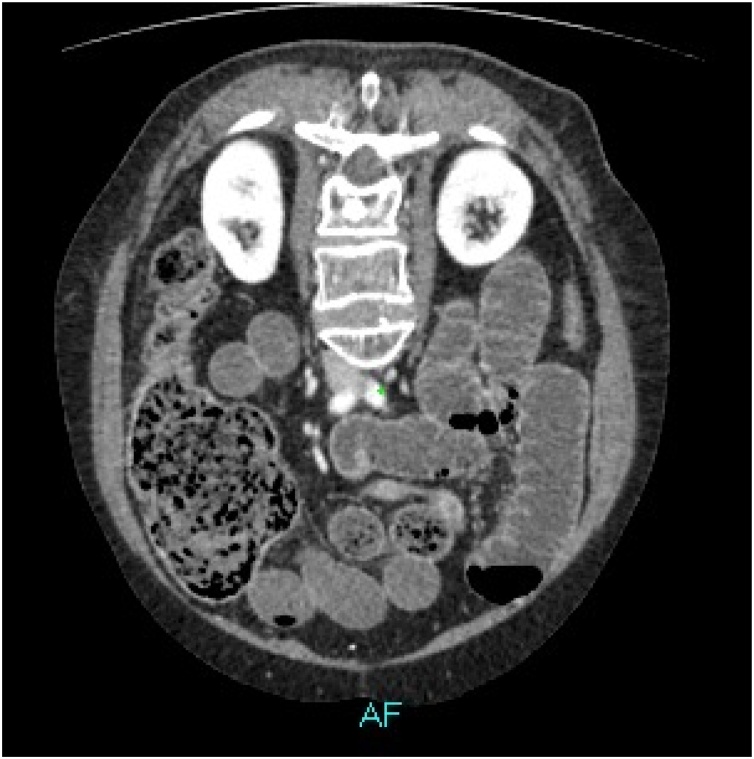
Axial oblique view.

CT scan with IV contrast demonstrated marked diffuse dilatation of small bowel loop and the cecum with abrupt point of transition at the junction of cecum and the ascending colon. Mild ascites was seen in the peri-hepatic region, in the pericecal region and the pelvis (Fig. 3, Fig. 4).

Based on radiology, no proper cause of intestinal obstruction could be identified. The patient was offered colonoscopy before laparotomy but she chose to get operated. Surgery was performed by consultant surgeon and senior registrar. Patient was counselled for right hemicolectomy, diversion and stoma formation.

**Fig. 5:**
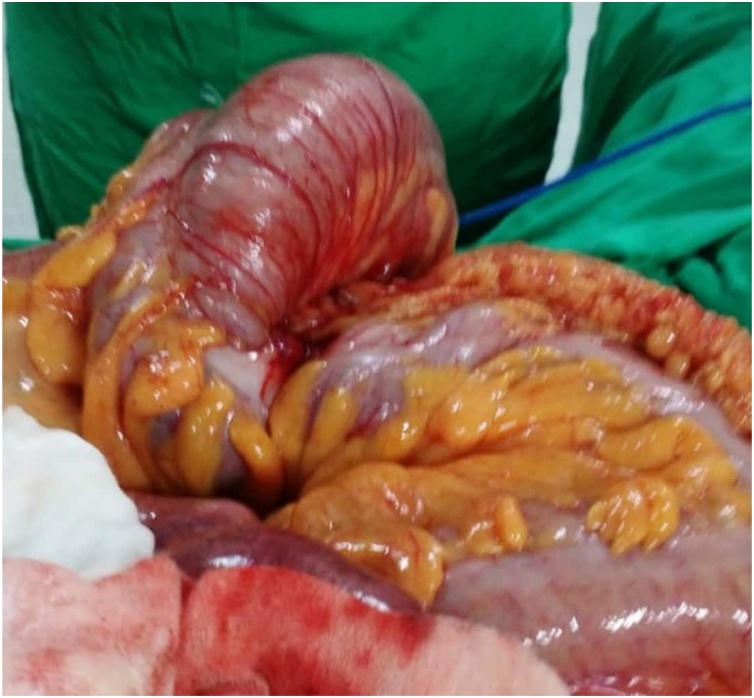
Intra-op cecal bascule.

On laparotomy, around 200 ml reactionary fluid was aspirated. The cecum and small bowel were distended but healthy looking. Cecum was found rotated anteriorly upon itself (Fig. 5). Distal to the caecum, the large bowel was collapsed. There was no other obvious pathology observed. Liver and peritoneum were normal. Cecectomy was done with end to end ileo-colic anastomosis.

**Fig. 6:**
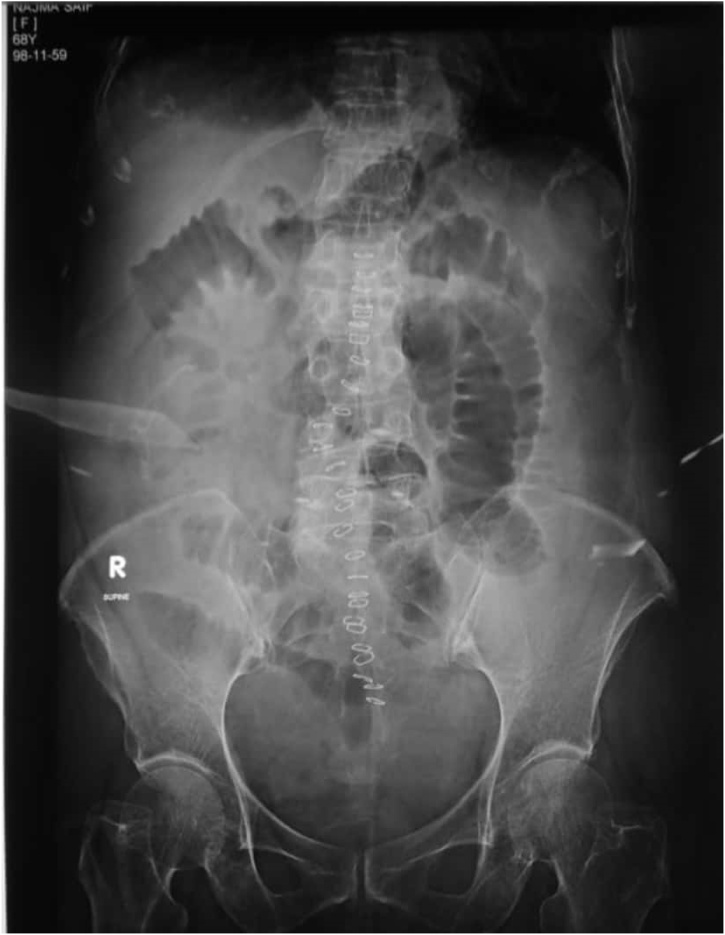
X-ray abdomen supine.

Post operatively, the patient was kept in the general ward, and she developed ileus (Fig. 6). She was given chewing gum 2nd post op day, and allowed sips 3rd post op day. Her potassium was replaced, glycerin suppositories given. Her ileus resolved on 7th post op day and she was discharged the next day.

Patient was called for follow-up after two weeks in which she reported uneventful post discharge recovery.

## 3 Discussion

Cecal bascule is a rare form of cecal volvulus, where the cecum folds anteriorly instead of twisting upon its axis [6]. This results in a deep crease which acts as a functional transit point. Due to a competent ileocecal valve this becomes a closed-loop and confines distension only to cecum [7]. The development of a cecal bascule is dependent upon hypermobility of the cecum [8]. It has been reported that adhesions formed may fix the anterior cecal wall to the anterior wall of ascending colon leading to cecal bascule [9,10]. This may be after a surgical procedure, such as Laparoscopic cholecystectomy [6], laparoscopic hernia repair [11], laparoscopic gastric bypass [12] or pelvic surgery such as total abdominal hysterectomy [13]. It has also been reported in a child who had a Nissen fundoplication [7]. Another hypothesis states that changes in the patient position during surgeries from reverse Tendelenburg to a horizontal plane may allow a hypermobile cecum to fold anteriorly and obstruct the large bowel causing cecal bascule [7]. It is agreed upon that for the development of cecal bascule, the presence of a competent ileocecal valve is required regardless of the accompanying adhesions [5,13].

The term embryogenic mobile cecum is used to define congenital mal-fixation of cecum to the right iliac fossa. This is due to a long mesentery which is fixed to the retroperitoneum by a narrow base of origin or the inability of right colonic mesentery to fuse to the peritoneum [6].

Cecal bascule is difficult to diagnose and maybe misdiagnosed as recurrent appendicitis until a severe attack ensues [14]. Unlike the “coffee bean” sign which occurs in sigmoid volvulus, there is no pathognomic sign for cecal bascule on a plain x-ray, resulting in an interpretation of non-specific, abnormal gas pattern [7]. CT may clearly define the large bowel anatomy but in our case it was non-diagnostic.

The treatment of choice for ceacal bascule is surgery. Simple reduction can lead to recurrence, and hence cecopexy or resection and anastomosis should be the preferred approach [7].

## 4 Conclusion

Cecal bascule is a rarity in surgical patients. Due to its non-specific radiology, it may be undiagnosed. A CT scan may or may not prove to be a useful modality. In patients who have undergone surgery before, this diagnosis should be placed in the differential. Surgical aim should be to prevent recurrence. Cecopexy or resection should be preferred.

## Declaration of Competing Interest

The authors report no declarations of interest.

## Sources of funding

No funding.

## Ethical approval

Study exempt from ethical approval.

## Consent

Written informed consent was obtained from the patient for publication of this case report and accompanying images. A copy of the written consent is available for review by the Editor-in-Chief of this journal on request.

## Author contribution

Dr. S. Areesha Shakeel: Data curation, Writing - Original draft preparation.

Dr. Junaid Zaman: Supervision.

Dr. Hamza Haroon: Writing - Reviewing and Editing.

## Registration of research studies

Not applicable.

## Guarantor

Dr. Syeda Areesha Shakeel.

## Provenance and peer review

Not commissioned, externally peer-reviewed.

## CRediT authorship contribution statement

**S. Areesha Shakeel:** Data curation, Writing - original draft. **Junaid Zaman:** Supervision. **Hamza Haroon:** Writing - review & editing.
